# What predicts health facility delivery among women? analysis from the 2021 Madagascar Demographic and Health Survey

**DOI:** 10.1186/s12884-024-06252-1

**Published:** 2024-02-07

**Authors:** Ebenezer Kwesi Armah-Ansah, Eugene Budu, Elvis Ato Wilson, Kenneth Fosu Oteng, Nhyira Owusuaa Gyawu, Bright Opoku Ahinkorah, Edward Kwabena Ameyaw

**Affiliations:** 1 Department of Population and Development, National Research University - Higher School of Economics, Moscow, Russia; 2 Population Dynamics Sexual and Reproductive Health Unit, African Population and Health Research Center, Nairobi, Kenya; 3 Department of Population and Health, University of Cape Coast, Cape Coast, Ghana; 4 Korle Bu Teaching Hospital, P. O. Box, 77, Accra, Ghana; 5 Kintampo Health Research Centre, Ghana Health Service, Kintampo, Ghana; 6 Ashanti Regional Health Directorate, Ghana Health Service, Kumasi, Ghana; 7 Quality management Unit, Korle Bu Teaching Hospital, P. O. Box, 77, Accra, Ghana; 8 School of Clinical Medicine, University of New South Wales Sydney, Sydney, Australia; 9 Institute of Policy Studies and School of Graduate Studies, Lingnan University, Tuen Mun, Hong Kong; 10 L & E Research Consult Ltd, Upper West Region, Wa, Ghana

**Keywords:** Madagascar, Demographic and Health Survey (DHS), Health facility, Delivery, Universal health coverage, Health insurance

## Abstract

**Background:**

One of the pivotal determinants of maternal and neonatal health outcomes hinges on the choice of place of delivery. However, the decision to give birth within the confines of a health facility is shaped by a complex interplay of sociodemographic, economic, cultural, and healthcare system-related factors. This study examined the predictors of health facility delivery among women in Madagascar.

**Methods:**

We used data from the 2021 Madagascar Demographic and Health Survey. A total of 9,315 women who had a health facility delivery or delivered elsewhere for the most recent live birth preceding the survey were considered in this analysis. Descriptive analysis, and multilevel regression were carried out to determine the prevalence and factors associated with health facility delivery. The results were presented as frequencies, percentages, crude odds ratios and adjusted odds ratios (aORs) with corresponding 95% confidence intervals (CIs), and a p-value < 0.05 was used to declare statistical significance.

**Results:**

The prevalence of health facility delivery was 41.2% [95% CI: 38.9–43.5%]. In the multilevel analysis, women aged 45–49 [aOR = 2.14, 95% CI = 1.34–3.43], those with secondary/higher education [aOR = 1.62, 95% CI = 1.30–2.01], widowed [aOR = 2.25, 95% CI = 1.43–3.58], and those exposed to mass media [aOR = 1.18, 95% CI = 1.00-1.39] had higher odds of delivering in health facilities compared to those aged 15-49, those with no formal education, women who had never been in union and not exposed to mass media respectively. Women with at least an antenatal care visit [aOR = 6.95, 95% CI = 4.95–9.77], those in the richest wealth index [aOR = 2.74, 95% CI = 1.99–3.77], and women who considered distance to health facility as not a big problem [aOR = 1.28, 95% CI = 1.09–1.50] were more likely to deliver in health facilities compared to those who had no antenatal care visit. Women who lived in communities with high literacy levels [aOR = 1.54, 95% CI = 1.15–2.08], and women who lived in communities with high socioeconomic status [aOR = 1.72, 95% CI = 1.28–2.31] had increased odds of health facility delivery compared to those with low literacy levels and in communities with low socioeconomic status respectively.

**Conclusion:**

The prevalence of health facility delivery among women in Madagascar is low in this study. The findings of this study call on stakeholders and the government to strengthen the healthcare system of Madagascar using the framework for universal health coverage. There is also the need to implement programmes and interventions geared towards increasing health facility delivery among adolescent girls and young women, women with no formal education, and those not exposed to media. Also, consideration should be made to provide free maternal health care and a health insurance scheme that can be accessed by women in the poorest wealth index. Health facilities should be provided at places where women have challenges with distance to other health facilities. Education on the importance of antenatal care visits should also be encouraged, especially among women with low literacy levels and in communities with low socioeconomic status.

## Introduction

A profound event that significantly has far-reaching implications for the health and well-being of women is childbirth. According to estimates, over 40% of all pregnancies may experience some form of complication [1]. The availability of emergency obstetric treatment inside healthcare facilities and the presence of trained birth attendants (TBAs) are acknowledged as critical components in lowering the incidence of maternal and neonatal mortality rates globally. Therefore, it is most convenient for a woman to give birth in a health facility where any problems that may arise are quickly resolved [1–6].

Maternal and child health is a major concern in many low- and middle-income countries (LMICs), with a focused commitment to enhancing access to skilled birth attendance and reducing maternal and neonatal mortality rates [7]. One of the pivotal determinants of maternal and neonatal health outcomes hinges on the choice of place of delivery [8]. However, many women in the reproductive age in LMICs continue to experience challenges in obtaining and using maternal healthcare services such as pregnancy and delivery. As a result, they choose to give birth at home with the help of a TBA rather than in a health facility under the supervision of a skilled health professional [9, 10].

Health facility delivery, often overseen by skilled birth attendants, is considered the cornerstone of safe motherhood practices globally [2–4]. This choice ensures access to timely medical interventions capable of addressing complications during childbirth, thereby reducing the risks of maternal and neonatal mortality. However, the decision to give birth at a healthcare facility is determined by a complex interaction of sociodemographic, economic, cultural, and healthcare system-related variables [3, 5, 6].

Over the last two decades, Madagascar’s maternal mortality ratio (MMR) has dropped from 658 to 392 deaths per 100,000 live births [11, 12]. Previous studies and surveys, however, have highlighted obstetric issues caused by home births supported by TBAs, low health insurance subscriptions, and insufficient medical staff and equipment as the major reasons for the high MMR in Madagascar [13]. As a result, maternal mortality in Madagascar continues to be a public health issue [14]. Access to health care facilities, on the other hand, remains a serious challenge in Madagascar, where health staff are unevenly dispersed and the majority of people live in extremely rural and difficult-to-reach locations with poor road and communication networks [15].

This study examined the predictors of health facility delivery in Madagascar, drawing from the wealth of data painstakingly collected through the Madagascar Demographic and Health Survey (MDHS). By delving into the intricate web of factors underpinning this pivotal choice, this research seeks to contribute significantly to the discourse on maternal and child health within the context of Madagascar. Furthermore, this study will provide key evidence that can be used by policymakers and program managers to design and implement interventions tailored to increasing the prevalence of health facility deliveries and ameliorating overall maternal and neonatal outcomes.

## Materials and methods

### Study area

Madagascar is the fifth-largest island, situated on the southeastern coast of sub-Saharan Africa (SSA), with a land area of over 587,041 square kilometers. Antananarivo is the capital city, and the country had a population of about 28 million as of 2020. Madagascar has a life expectancy of 63 years and 68 years for men and women, respectively [16]. Madagascar has witnessed a decline in fertility, from 6.1 children per woman in 1992 to 4.3 children per woman in 2021. Forty-seven (47) children out of 1,000 live births die before their first birthday, and 1 in 13 dies before reaching age 5 [17].

As of 2019, Madagascar’s healthcare was under four different types of delivery systems: basic health centres; district hospitals; regional hospitals; and university hospitals. There were 824 and 124 private health dispensary facilities and clinics respectively; 22 university hospitals; 99 district hospitals; 16 regional hospitals; and 2,710 basic health centers. The Ministry of Public Health is the central coordinator of health services; however, there are regional and district health offices that implement and supervise health programmes [18, 19]. The health system in Madagascar is largely dependent on donor funding from the United Nations International Children’s Emergency Fund (UNICEF), the World Health Organization (WHO), the United Nations Population Fund (UNFPA), and the United States Agency for International Development (USAID). Hence, it is challenged with equitable healthcare financing and access, a robust health information system, and research for efficient planning [20].

### Data source and study population

**Fig. 1:**
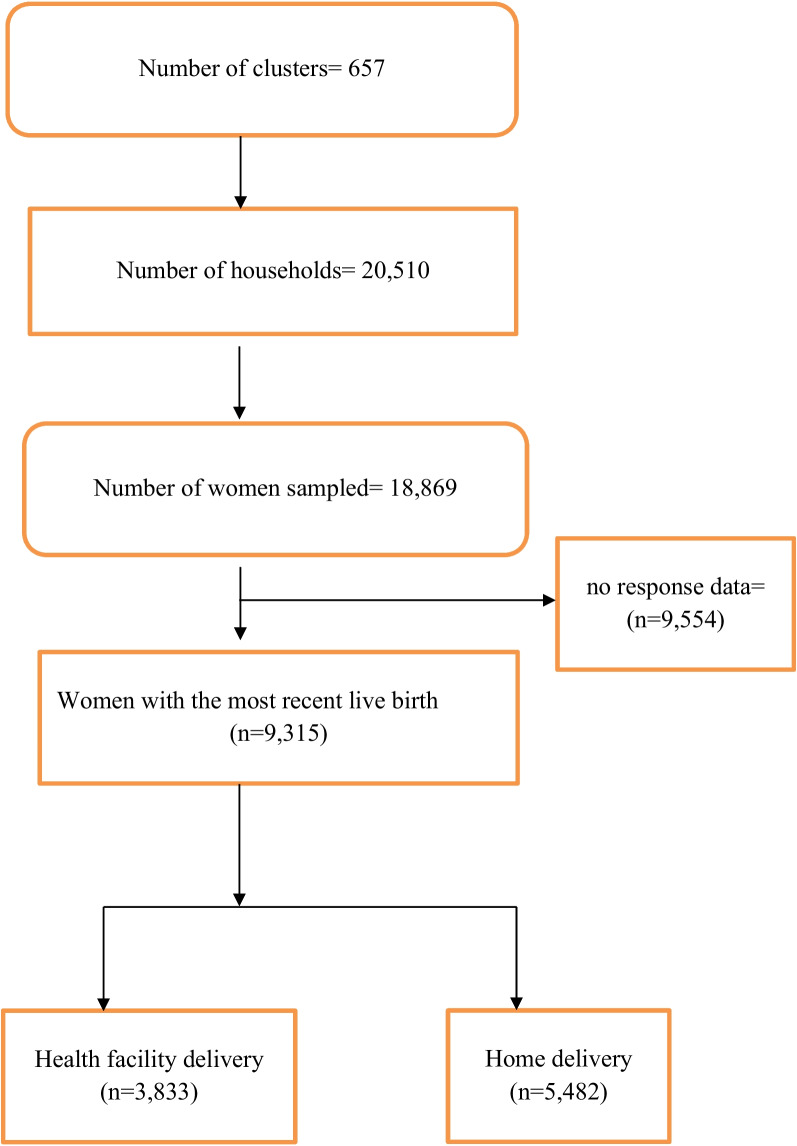
Sampling procedure

This study used data from the 2021 MDHS. The MDHS, conducted periodically, offers an invaluable repository of data for in-depth investigation into this critical facet of reproductive healthcare within the country. The National Institute of Statistics (INSTAT), in collaboration with the Ministry of Public Health, performed the fifth edition of the MDHS in 2021. It monitors and assesses national development plans and programmes, as well as the Sustainable Development Goals (SDGs) [17]. The DHS is a five-year nationally representative study undertaken in a number of LMICs. Through interviews with women of reproductive age (15–49 years), it focuses primarily on maternal and child health. From March through July of 2021, this data were acquired using a stratified sampling technique. The overall sample size for this survey was 18,869 women aged 15–49 from 20,510 homes who were present the night before the survey. The MDHS sampling method is well documented elsewhere [17]. The MDHS uses standardised techniques for sampling, questionnaire development, data collection, cleaning, coding, and analysis. Details of the methodology, instruments, instrument pretesting, training, and recruitment of enumerators are recorded in the 2021 MDHS final report [17]. The dataset may be downloaded for free at: https://dhsprogram.com/data/dataset/Madagascar_Standard-DHS_2021.cfm?flag=1. Figure 1 below is a framework showing the sampling procedure for selecting the study participants.

### Description of variables

#### Dependent variables

The dependent variable was whether a woman had a health facility delivery or delivered elsewhere for the most recent live birth preceding the 2021 MDHS. As the outcome variable, we recoded “place of delivery” as either “health facility delivery” = ‘1’ (when the birth occurred at a hospital, health center, or health post) or “home delivery = ‘0’ (where the birth occurred at the respondent’s home or any other place). Women who replied “other” were not included in the analysis.

### Conceptual framework for health facility delivery

In order to select variables that are associated health facility delivery among women in Madagascar, the authors adapted the Andersen’s Framework of Healthcare Services Utilisation Model. The authors used the fourth version of the framework that was developed in 1990 [21]. This model postulates that three factors influence a woman to consider the use of a healthcare service. These characteristics are (1) predisposing factors; (2) enabling factors; and (3) need for care factors [21].

**Fig. 2:**
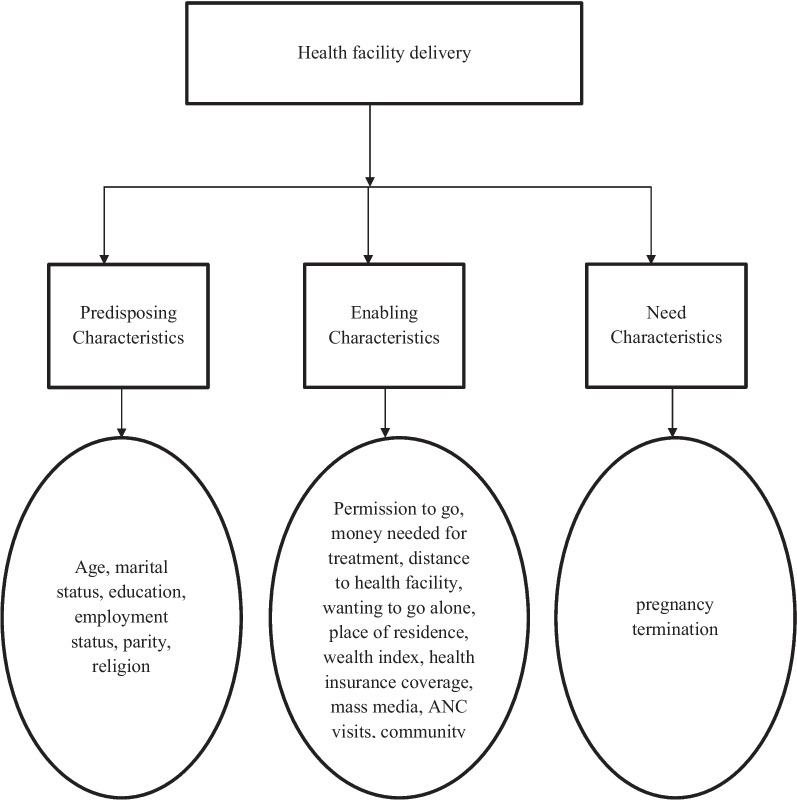
Conceptual Framework

The predisposing factors are the characteristics that promote or impede the use of a healthcare service [22]. These characteristics are social structures, health beliefs, and demographics, including age, education, occupation, parity, religion, and other factors [21]. Another characteristic proposed in this model is the enabling characteristic, which is related to the logistical aspects that influence the use of a healthcare service. These characteristics include wealth, health insurance coverage, access to healthcare, place of residence, and other factors [21]. The need for care factors are the last characteristic that is proposed in the fourth phase of the model. The need for care factors are the most immediate cause of health service use [21]. Based on studies on existing literature on health facility delivery [23–28], this model was used to select the factors associated with health facility delivery. Figure 2 below is the conceptual framework adapted from Andersen’s healthcare services utilisation model.

#### Independent variables

Age, education, marital status, employment status, religion, terminated pregnancy, parity, antenatal care (ANC) visits, sex of household head, health insurance coverage, wealth index, and mass media were included in the analysis. Other variables include permission to go, problem with money needed for treatment, problem with distance to health facility, problem with wanting to go alone, place of residence, community literacy level, and community socioeconomic status.

### Operational definitions

#### Mass media

Mass media included listening to radio, watching television, and reading newspapers and magazines. These three variables had the same response options: “not at all”, “less than once a week”, and “at least once a week”. Based on the literature, we grouped the response options into “no,” which meant no mass media exposure (not at all), and “yes,” which meant mass media exposure (less than once a week and at least once a week) [29, 30].

#### Community literacy level

The proportion of women aged 15–49 who could either read and write only.

#### Community socioeconomic status

Community socioeconomic status was assessed based on household wealth. We utilized principal component analysis to assess the number of women who were in the richest wealth quintile. A standardized score was established with a mean score of 0 and a standard deviation. The scores were then split into three tertiles: 1 (least disadvantaged), 2 (middle disadvantaged), and 3 (most disadvantaged), with tertile 1 representing a better socioeconomic position and tertile 3 representing a worse socioeconomic position [31].

### Statistical analysis

Stata version 14.2 was used to analyse the data. Descriptive, bivariate, and multilevel regression analyses were performed. The descriptive analysis was performed to describe the study sample. In the bivariate analysis, Pearson’s chi-square (X^2^) test was used to evaluate the relationships between health facility delivery and each of the study’s independent variables. Statistically significant variables in the bivariate analysis were moved to the multilevel regression model. Adjusted odds ratios (aORs) with 95% confidence intervals (CIs) were used to present the results of the multilevel regression. There was no evidence of collinearity among the explanatory variables, according to the variance inflation factor (VIF) multicollinearity test (mean VIF = 1.54, maximum VIF = 2.66, minimum VIF = 1.02).

Four models were constructed in Table 3. The first model (Model 0) was the empty model, which had no explanatory variable but showed the variance of the outcome variable attributable to the distribution of the primary sampling units. The second model contained only the individual/household-level factors (Model 1), while Model 2 had only the community-level factors. The final model (Model 3) was a complete model that had both individual/household and community-level factors. The Stata command ‘melogit’ was used in fitting these models. Model comparison was also done using the Akaike’s Information Criterion (AIC) test. The study sample was weighted and the survey set command was used in the analyses to account for the survey’s complex nature and the generalizability of the findings.

## Results

### Prevalence of health facility delivery

**Fig. 3:**
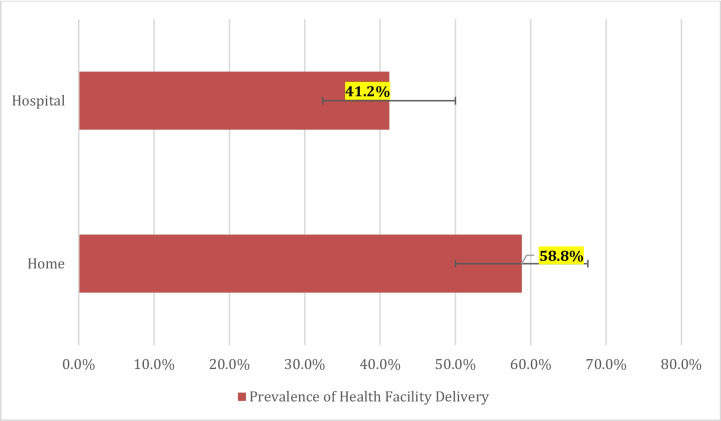
Prevalence of health facility delivery, 2021 MDHS

The prevalence of health facility delivery among women in Madagascar was 41.2% [95% CI: 38.9–43.5%] (see Fig. 3).

### Description of the study sample

In Table 1, we realized majority of the women (27.2%) were aged 20–24 while almost half of the women (43.9%) had primary education. Almost two-thirds (66.4%) were married women whereas about four in five (86.3%) of the women were working. Close to a third (33.1%) had four or more births, while more than half (57.7%) were exposed to mass media. About four in five women (89.2%) have had at least an ANC visit, and 97.2% were not covered by health insurance. A higher proportion (84.5% and 70.9%) of the women resided in the rural areas and were Christians respectively whereas most of the women (22.5%, 84.7%, 64.3%, 69.4%) were in the poorest wealth quintile, had not a big problem asking for permission to go, distance to health facility and wanting to go alone respectively. However, almost six in ten of the women (59.2%) had a big problem with money needed for treatment. More than half (55.9%) of the women were in low community socioeconomic status whereas more than a third (35.7%) were in medium community literacy level.

**Table 1:** Description of study sample (Weighted, *N* = 9,315)

**Variables **	**Weighted N**	**Percentage (%)**
**Individual/household-level variables**		
**Age **		
15-19	1,116	11.9
20-24	2,534	27.2
25-29	2,149	23.1
30-34	1,613	17.3
35-39	1,106	11.9
40-44	597	6.4
45-49	200	2.2
**Education **		
No education	1,852	19.9
Primary	4,086	43.9
Secondary/higher	3,377	36.2
**Marital Status**		
Never in union	769	8.3
Married	6,190	66.4
Cohabitation	1,160	12.5
Widowed	114	1.2
Divorced	1,082	11.6
**Employment status**		
Not working	1,278	13.7
working	8,037	86.3
**Parity**		
One birth	2,614	28.0
Two births	2,116	22.7
Three births	1,505	16.2
Four or more births	3,080	33.1
**Mass Media**		
No	3,943	42.3
Yes	5,372	57.7
**ANC visits**		
No	1,008	10.8
1 or more	8,307	89.2
**Health insurance coverage**		
No	9,054	97.2
Yes	261	2.8
**Religion**		
Christianity	6,600	70.9
Islam	87	0.9
Traditional	236	2.5
No religion	2,392	25.7
**Wealth index**		
Poorest	2,096	22.5
Poorer	1,942	20.9
Middle	1,895	20.3
Richer	1,818	19.5
Richest	1,564	16.8
**Terminated Pregnancy**		
No	8,077	86.7
Yes	1,238	13.3
**Community-level variables**		
**Place of residence**		
Urban	1,548	16.6
Rural	7,767	83.4
Problem with **permission to go to the health facility**		
Big problem	1,423	15.3
Not a big problem	7,892	84.7
Problem with **money needed for treatment**		
Big problem	5,518	59.2
Not a big problem	3,797	40.8
Problem with **distance to a health facility**		
Big problem	3,326	35.7
Not a big problem	5,989	64.3
Problem with **not wanting to go to the health facility alone**		
Big problem	2,847	30.6
Not a big problem	6,468	69.4
**Community literacy level**		
Low	2,819	30.3
Medium	3,325	35.7
High	3,171	34.0
**Community socioeconomic status**		
Low	5,204	55.9
Moderate	1,074	11.5
High	3,037	32.6

###  Distribution of the independent variables accross health facility delivery

In the chi-square test results (Table 2), we realized that a higher proportion of women aged 30–34 (43.3%) had health facility delivery. Health facility delivery was highest among women with secondary or higher education (60.4%) and widowed women (44.9%). Not-working women (49.8%), women with one birth (51.8%), and women exposed to mass media (51.8%) had the highest proportion of health facility deliveries. A higher proportion of women with at least an ANC visit (45.4%) and health insurance coverage (81.6%) had health facility delivery compared with those with no ANC visit (6.4%) and no health insurance coverage (40.0%). Women who belonged to the richest wealth quintile (72.6%) and Muslims (56.8%) had the highest proportion of health facility deliveries. Women who resided in urban areas (61.2%), had ever terminated pregnancy (44.3%), had no big problem asking permission to go to the health facility (41.9%), had no big problem with the money needed for treatment (47.9%), had no big with the distance to a health facility (48.1%), and did), not have a big problem with not wanting to go alone (45.6%). Women with high community literacy levels (58.9%) and high community socioeconomic status (63.7%) had the highest proportion of health facility deliveries.

**Table 2:** Bivariate results on the factors associated with health facility delivery (Weighted, *N* = 9,315)

**Variables **	**Health facility delivery (%)**	**χ2**	***p*-value**
**Individual/Household-level variables**			
** Age **		**18.46**	**0.005**
15-19	39.7		
20-24	40.8		
25-29	42.7		
30-34	43.3		
35-39	41.7		
40-44	34.9		
45-49	36.4		
**Education **		**971.31**	**<0.001**
No education	20.4		
Primary	34.7		
Secondary/higher	60.4		
**Marital Status**		**52.71**	**<0.001**
Never in union	40.6		
Married	43.4		
Cohabitation	33.0		
Widowed	44.9		
Divorced	36.9		
**Employment status**		**37.84**	**<0.001**
Not working	49.8		
working	39.8		
**Parity**		**264.76**	**<0.001**
One birth	51.8		
Two births	44.2		
Three births	40.8		
Four or more births	30.2		
**Mass Media**		**605.14**	**<0.001**
No	26.7		
Yes	51.8		
**ANC visits**		**635.82**	**<0.001**
No	6.4		
1 or more	45.4		
**Health insurance coverage**		**177.13**	**<0.001**
No	40.0		
Yes	81.6		
**Religion**		**583.96**	**<0.001**
Christianity	48.5		
Islam	56.8		
Traditional	19.6		
No religion	22.4		
**Wealth index**		**6.53**	**<0.001**
Poorest	19.4		
Poorer	31.1		
Middle	38.4		
Richer	52.7		
Richest	72.6		
**Terminated Pregnancy**		**5.99**	**0.014**
No	40.7		
Yes	44.3		
**Community-level variables**			
**Place of residence**		**485.41**	**<0.001**
Urban	61.2		
Rural	37.2		
**Problem with permission to go to the health facility**		**9.14**	**0.003**
Big problem	36.8		
Not a big problem	41.9		
**Problem with money need for treatment**		**107.64**	**<0.001**
Big problem	36.9		
Not a big problem	47.9		
**Problem with distance to health facility**		**322.01**	**<0.001**
Not a big problem	28.6		
Not a big problem	48.1		
**Problem with not wanting to go to the health facility alone**		**180.81**	**<0.001**
Not a big problem	31.1		
Not a big problem	45.6		
**Community literacy level**		**853.24**	**<0.001**
Low	22.5		
Medium	40.1		
High	58.9		
**Community socioeconomic status**		**5.72**	**<0.001**
Low	28.9		
Moderate	37.3		
High	63.7		

*p*-value less than 0.05 indicates statistical significance

In Table 2, it was found that age (years) (*p* = 0.005), educational level (*p* <0.001), marital status (*p*<0.001), employment status (*p*<0.001), parity (*p*<0.001), mass media (*p*<0.001), ANC visits (*p*<0.001), health insurance coverage (*p*<0.001), religion (*p*<0.001), wealth (*p*<0.001), terminated pregnancy (*p* = 0.014), place of residence (*p*<0.001), problem with permission to go to the health facility (*p* = 0.003), problem with money needed for treatment (*p*<0.001), problem with distance to health facility (*p*<0.001), problem with not wanting to go to the health facility alone (*p*<0.001), community literacy level (*p*<0.001), and community socioeconomic status (*p*<0.001) were associated with health facility delivery in Madagascar.

### Multilevel regression results of individual/household and community-level factors associated with health facility delivery

As shown in Model 3 of Table 3, women aged 45–49 [aOR = 2.14, 95% CI = 1.34–3.43], women with secondary or higher education [aOR = 1.62, 95% CI = 1.30–2.11], and those widowed [aOR = 2.25, 95% CI = 1.43–3.55], were more likely to have health facility delivery compared to women aged 15–19, those with no formal education, and never in union women, respectively. Women with four or more births [aOR = 0.38, 95% CI = 0.29–0.51] had lower odds of having health facility delivery as compared with those with one birth. The odds of having health facility delivery were higher among women with at least an ANC visit (aOR = 6.95, 95% CI = 4.95–9.77), compared to women with no ANC visit, while those who were exposed to mass media [aOR = 1.18, 95% CI = 1.00–1.39] had higher odds of having health facility delivery compared to women who had no mass media exposure.

**Table 3:** Multilevel regression results of individual/household and community-level factors associated with health facility delivery

**Variables**	**Model 0**	**Model 1** **aOR (95% CI)**	**Model 2** **aOR (95% CI)**	**Model 3** **aOR (95% CI)**
**Fixed effect**				
** Individual/household-level variables**				
** Age**				
15–19		Ref		Ref
20–24		0.89 (0.71–1.12)		0.88 (0.70–1.10)
25–29		1.21 (0.94–1.55)		1.19 (0.92–1.52)
30–34		1.58** (1.18–2.10)		1.53** (1.14–2.04)
35–39		1.80** (1.29–2.52)		1.73** (1.24–2.42)
40–44		1.51* (1.03–2.20)		1.44 (0.98–2.11)
45–49		2.13** (1.33–3.42)		2.14** (1.34–3.43)
** Education**				
No education		Ref		Ref
Primary		1.19 (0.98–1.44)		1.15 (0.95–1.39)
Secondary/higher		1.72*** (1.38–2.13)		1.62*** (1.30–2.01)
** Marital Status**				
Never in union		Ref		Ref
Married		1.33** (1.07–1.65)		1.32** (1.06–1.64)
Cohabitation		1.28 (0.96–1.71)		1.28 (0.96–1.70)
Widowed		2.34*** (1.49–3.67)		2.25*** (1.43–3.55)
Divorced		1.37* (1.05–1.80)		1.34* (1.02–1.76)
** Employment status**				
Not working		Ref		Ref
Working		0.84 (0.69–1.04)		0.86 (0.70–1.06)
** Parity**				
One birth		Ref		Ref
Two births		0.61** (0.50–0.75)		0.61** (0.50–0.75)
Three births		0.48*** (0.37–0.62)		0.49*** (0.38–0.63)
Four or more births		0.37*** (0.28–0.49)		0.38*** (0.29–0.51)
** Mass media**				
No		Ref		Ref
** Yes**		1.21* (1.03–1.42)		1.18* (1.00-1.39)
** ANC visit**				
** No**		Ref		Ref
** 1 or more**		7.07*** (5.02–9.96)		6.95*** (4.95–9.77)
** Health insurance coverage**				
No		Ref		Ref
Yes		1.67 (0.98–2.84)		1.55 (0.91–2.63)
** Religion**				
Christianity		Ref		Ref
Islam		0.94 (0.53–1.68)		0.92 (0.52–1.64)
Traditional		0.54** (0.34–0.85)		0.56* (0.35–0.89)
No/other		0.68*** (0.55–0.83)		0.73** (0.59–0.90)
** Terminated Pregnancy**				
No		Ref		Ref
Yes		0.97 (0.81–1.15)		0.98 (0.82–1.16)
** Wealth index**				
Poorest		Ref		Ref
Poorer		1.62*** (1.32–1.99)		1.53*** (1.25–1.87)
Middle		1.72*** (1.37–2.15)		1.53*** (1.22–1.92)
Richer		2.50*** (1.95–3.20)		2.01*** (1.55–2.60)
Richest		3.97*** (3.00-5.26)		2.74*** (1.99–3.77)
**Community-level variables**				
** Problem with permission to go to the health facility**				
Big problem			Ref	Ref
Not a big problem			0.94 (0.78–1.13)	0.94 (0.78–1.13)
** Problem with money needed for treatment**				
Big problem			Ref	Ref
Not a big problem			1.27*** (1.11–1.45)	1.05 (0.91–1.21)
** Problem with distance to health facility**				
Big problem			Ref	Ref
Not a big problem			1.22* (1.04–1.43)	1.28** (1.09–1.50)
** Problem with not wanting to go to the health facility alone**				
Big problem			Ref	Ref
Not a big problem			1.11 (0.94–1.31)	1.10 (0.92–1.32)
** Place of residence**				
Urban			Ref	Ref
Rural			0.86 (0.66–1.14)	1.17 (0.88–1.56)
** Community literacy level**				
Low			Ref	Ref
Medium			1.86** (1.44–2.41)	1.16 (0.89–1.50)
High			3.18*** (2.38–4.24)	1.54** (1.15–2.08)
** Community socioeconomic status**				
Low			Ref	Ref
Moderate			1.26 (0.87–1.82)	0.98 (0.68–1.40)
High			3.13*** (2.35–4.16)	1.72*** (1.28–2.31)
** Random effect results**				
PSU variance (95% CI)	2.14 (1.83–2.50)	0.97 (0.80–1.18)	1.01 (0.83–1.22)	0.89 (0.73–1.09)
ICC	39.4	22.8	23.5	21.3
Wald chi-square	Ref	721.85***	418.31***	920.90***
** Model fitness**				
AIC	10,769.68	9,760.38	10,333.45	9,709.52
BIC	10,783.96	9,967.43	10,411.98	9,980.82
**N**	9,315	9,315	9,315	9,315
**Number of groups**	649	649	649	649

Source: 2021 MDHS

Ref-Reference category, *p* < 0.05, ^**^p < 0.01, ^***^p < 0.001, aOR adjusted odds ratio

*PSU* Primary Sampling Unit, *ICC* Intra-Class Correlation, *aOR* adjusted odds ratio, *AIC* Akaike’s Information Criterion, *BIC* Bayesian Information Criterion

^*^p < 0.05, ^**^p < 0.01, ^***^p < 0.001

Women who considered distance to health facilities as not a big problem [aOR = 1.28, 95% CI = 0.29–0.51], had higher odds of having health facility delivery as compared with those who considered that as a big problem. Regarding religion, women affiliated with traditional religion [OR = 0.56, 95% CI = 0.35–0.89] had the lowest likelihood of having health facility delivery. Women in the richest wealth index [aOR = 2.74, 95% CI = 1.99–3.77], women who lived in communities with high literacy levels index [aOR = 1.54, 95% CI = 1.15–2.08], and those who lived in communities with high socioeconomic status index [aOR = 1.72, 95% CI = 1.28–2.31], had higher odds of having health facility delivery compared to those in the poorest wealth index, low community literacy levels, and low community socioeconomic status, respectively.

### Random effects (measures of variation) results

As shown in Table 3, the AIC values show that there was a decline in models 1 and 2, which had individual/household-level variables and community-level variables, respectively, compared to the final model (model 3). This substantial decrease in the models supports the goodness of fit of model 3, which had individual, household, and community-level variables. Model 3 is the complete model that was selected for predicting the factors associated with health facility delivery among women in Madagascar. In the empty model, there were substantial variations in the likelihood of factors associated with health facility delivery across the clustering of the PSUs [σ2 = 2.14, 95% CI 1.83–2.50]. The ICC value for Model 0 shows that 39% of the variation in the place of delivery was attributed to the between-cluster variations of the characteristics. The variation between clusters then decreased to 22.8% in Model 1, which was the individual and household-level variables only. The ICC then increased to 23.5% in Model 2, which had only the community-level variables. In the final model (Model 3), the between-cluster variation then decreased to 21.3%. This can be attributed to the differences in the clustering of the PSUs, which account for the variations in the place of delivery.

## Discussion

Health facility delivery has been identified as one of the most effective methods for achieving the SDG which seeks to reduce maternal mortality ratio to less than 70 maternal deaths per 100,000 live births by 2030 [15, 32]. Globally, the prevalence of health facility delivery was 76% and 56% for SSA as of 2018 [33]. The prevalence of health facility delivery among women in Madagascar was found to be 41.2% in this analysis. This implies that the prevalence of health facility delivery is low among women in Madagascar. The prevalence found in this study is higher than the prevalence of 26.2% in Ethiopia [34], 28.7% in Bangladesh [35], and 41% in Nigeria [36]. However, the prevalence of health facility delivery in this study is lower than 66% in SSA [27], 82.7% in Southeast Ethiopia [37], and 87.4% in East Africa [28]. The possible explanation for the low prevalence of health facility delivery could be that Madagascar women may not be able access the majority of healthcare facilities due to distance and cost. Studies in Madagascar have revealed that less than 10% of women in Madagascar have health insurance, and more than half (51%) of health facilities in Madagascar have only a caregiver. Also, one in two health facilities is inaccessible year-round. Another explanation could be that 25.8% of women in Madagascar reside more than 5 km from the nearest healthcare facility, which is either understaffed or lacks sufficient medical care equipment and supplies [13, 38–41].

In the multilevel analysis, model 3 was the best fit for discussion. Model 3 had the lowest ICC of 21.3% and AIC of 9,709.52. The results from the multilevel analysis of this study were similar to previous studies conducted in Eritrea [10], SSA [27], Bangladesh [35], East Africa [28, 42, 43], south-Asian countries [44], and West Africa [45, 46].

The study revealed that individual-level variables including age, educational level, marital status, parity, ANC visits, mass media exposure, religion, and wealth were significantly associated with health facility delivery in Madagascar. Our study revealed that older women were more likely to choose health facility delivery as compared to younger mothers. This finding is in line with other studies conducted in Nigeria [36] and northern and south-central Ethiopia [47]. However, other studies conducted in northwest Ethiopia [48] and southern Ethiopia [49] were inconsistent with our study. One possible explanation is that older women are more aware of the obstetric complications associated with age and hence choose health facility delivery [47].

Several studies have shown that formal education influences women’s ability to make decisions about their reproductive health in SSA [27, 50, 51]. It was found that the odds of choosing a health facility delivery increase with an increase in women’s educational level. This finding is consistent with the findings of studies conducted in SSA [27], Ethiopia [42, 43], rural Ghana [45], Nepal [52], and Nigeria [4]. This reason could be attributed to the fact that formal education empowers and provides women with autonomy through the provision of essential information needed to deliver at a health facility during pregnancy. This essential information on reproductive health decisions safeguards the health of women and babies [27, 43, 50, 53].

Another important factor that influenced the choice of place of delivery is the marital status of women in Madagascar. It was found that women who were either married or had been married were more likely to utilize health facilities during delivery compared to women who were never in union. The finding is in line with previous studies conducted in East Africa [28, 54]. In contrast, studies conducted in southern Ethiopia [49] and Ghana [22] reported no statistically significant association between health facility delivery and marital status. The possible reason could be that women who are either married or have been married may receive spousal and family support in making health care decisions about maternal health service utilisation [54].

Irrespective of how many times women have given birth, they are advised to have their babies delivered in health facilities [32]. However, this current study found that the odds of health facility delivery women in Madagascar decrease with an increase in parity. The finding reveals that women with two or more births were less likely to opt for health facility delivery than those with one birth. This is consistent with studies conducted in Ghana [45], Uganda [55], and SSA [24, 26]. Studies have argued that primiparous women access health facilities more frequently because they are more susceptible to maternal complications during child delivery than multiparous women [56, 57]. Another plausible reason could be the financial burden associated with larger family sizes and the maternal experiences of women with more than one birth [24, 26].

Another important predictor of health facility delivery in Madagascar was antenatal care visits. It was found that women who had at least an ANC visit were more likely to utilize health facilities during delivery. The finding is in line with previous studies conducted in Asia [44, 52], Eastern Africa [34, 58], and SSA [24, 27, 59]. The reason could be that during ANC visits, women are most likely to be informed about the benefits associated with health facility delivery [43, 60].

The other most significant predictor of health facility delivery among women in Madagascar was mass media exposure. Women who were exposed to mass media had higher odds of health facility delivery than those who were not exposed to mass media. This finding was supported by previous studies conducted in SSA [27], Ghana [61, 62], and Ethiopia [63]. This reveals the positive influence of mass media on the choice of health facility delivery among women of reproductive age [27].

Religion also played an essential role for women in choosing a place of delivery [64]. Our finding, which shows that women affiliated with traditional religions had a lower likelihood of using health facilities during delivery compared to Christian women, confirms studies conducted in Ghana [22, 65]. Women who hold traditional and other beliefs may be less likely to give birth in a health facility due to their disapproval of contemporary medical procedures. These women may assume that pregnancy and labour are natural biological processes that do not need medical treatment until an emergency occurs [22].

Financial restrictions on access to and use of health care are pervasive in SSA, preventing many people, particularly the poor, from using health services [27]. In this current study, it was revealed that household wealth plays an important role in choosing the place of delivery during pregnancy. Consistent with previous studies conducted in Eastern Africa [28, 32, 34, 42], rural Ghana [45], and SSA [24, 26], this study confirms that health facility delivery among women in Madagascar increases with increasing wealth status. The plausible reason could be that the richest women can afford the necessary medical and transportation expenditures, which may improve their health-seeking behaviour and autonomy [34].

Furthermore, distance to health facilities, community literacy level, and community socioeconomic status were the community-level variables found to be significantly associated with women’s health facility delivery in Madagascar.

Another significant predictor of health facility delivery in this study was distance to the facility. The analysis revealed that distance to health facilities was not a big problem for women in Madagascar. This finding is consistent with studies conducted in the SSA [26] and East Africa [28]. This could be as a result of affordable and reliable transportation that can mitigate the impact of the distance [22]. It emphasizes how important it is to give the population access to maternal health care services [28].

The study revealed that community literacy level was an important determinant of health facility delivery among women in Madagascar. From the analysis, women who lived in communities with high literacy level in Madagascar were more likely to deliver in a health facility than their counterparts who lived in communities with low literacy level. The result from this current study is in line with a previous study conducted in SSA [24]. A plausible reason could be that educated women may have adequate material resources to access healthcare services [24].

The study found that community socioeconomic status has an effect on the choice of place of delivery. The finding was consistent with previous research conducted in SSA [24], Ghana [66], and Bangladesh [67], where women of high community socioeconomic status had higher odds of health facility delivery. This might be due to the availability of healthcare facilities within their range as well as their financial ability to obtain and use health care facilities [67].

### Strengths and limitations of the study

This study’s major strength is the use of current nationally representative data from the MDHS, which makes the study’s findings generalizable to women of reproductive age in Madagascar. Another strength is the rigorous analytical and statistical approach used to increase the dependability of our findings by estimating the cluster effect on health facility delivery. Despite these strengths, there are a few limitations inherent in this study. First, the research sample was confined to women of reproductive age (15–49) who had at least a birth five years prior to the survey. Moreover, the cross-sectional character of the MDHS and the causal-effect relationship could not be determined. Furthermore, recollection bias may affect survey participants’ self-reported data, which could lead to over- or under-reporting.

## Conclusion

The prevalence of health facility delivery in Madagascar is low in this current study. The Ministry of Public Health and its agencies ought to consider women’s age, women’s educational level, parity, marital status, ANC visits, mass media, religion, wealth, community literacy level, and community socioeconomic status when developing strategies to improve health facility delivery in Madagascar. The findings of this study call on stakeholders and the government to strengthen the health system of Madagascar using the framework for universal health coverage (UHC). There is also the need to implement programmes and interventions geared towards increasing health facility delivery among young adults, women with no formal education, and women with at least two births. Also, consideration should be made to provide free maternal health care and a health insurance scheme that can be accessed by women in the poorest wealth index. Finally, there is the need for further studies to consider involvement of family members in decision-making about place of delivery.

## Data Availability

Data is available on https://dhsprogram.com/data/dataset/Madagascar_Standard-DHS_2021.cfm?flag=1.

## Abbreviations

AIC: Akaike’s Information Criterion
ANC: Antenatal care
aOR: adjusted odds ratio
BIC: Bayesian Information Criterion
CI: confidence intervals
ICC: Intra-Class Correlation
LMICs: Low-and-middle-income countries
MDHS: Madagascar Demographic and Health Survey
MMR: Maternal Mortality Ratio
PSU: Primary Sampling Unit
Ref: Reference category
SDG: Sustainable Development Goal
SSA: Sub-Saharan Africa
VIF: Variance Inflation Factor
UHC: Universal health coverage
UNFPA: United Nations Population Fund
UNICEF: United Nations International Children’s Emergency Fund
USAID: United States Agency for International Development
WHO: World Health Organization
